# The Concept of Hormesis in Cancer Therapy – Is Less More?

**DOI:** 10.7759/cureus.261

**Published:** 2015-04-02

**Authors:** Andy Gaya, Charles A Akle, Satvinder Mudan, John Grange

**Affiliations:** 1 London Oncology Clinic, Guy's and St Thomas' NHS Foundation Trust; 2 Immodulon Therapeutics, The London Clinic; 3 Department of Academic Surgery, The Royal Marsden Hospital

**Keywords:** immunotherapy, low dose metronomic chemotherapy, stereotactic body radiation therapy, stereotactic ablative radiotherapy (sabr), cyberknife, hormesis, drug repositioning, inflammation, mtor

## Abstract

There has, in recent years, been a paradigm shift in our understanding of the role of the immune system in the development of cancers. Immune dysregulation, manifesting as chronic inflammation, not only facilitates the growth and spread of tumors but prevents the host from mounting effective immune defenses against it. Many attempts are being made to develop novel immunotherapeutic strategies, but there is growing evidence that a radical reevaluation of the mode of action of chemotherapeutic agents and ionizing radiation is required in the light of advances in immunology.

Based on the concept of hormesis – defined as the presence of different modes of action of therapeutic modalities at different doses – a ‘repositioning’ of chemotherapy and radiotherapy may be required in all aspects of cancer management. In the case of chemotherapy, this may involve a change from the maximum tolerated dose concept to low dose intermittent (‘metronomic’) therapy, whilst in radiation therapy, highly accurate stereotactic targeting enables ablative, antigen-releasing (immunogenic) doses of radiation to be delivered to the tumor with sparing of surrounding normal tissues. Coupled with emerging immunotherapeutic procedures, the future of cancer treatment may well lie in repositioned chemotherapy, radiotherapy, and more localized debulking surgery.

## Introduction and background

*“All things are poison, and nothing is without poison; only the dose permits something not to be poisonous”* – Paracelsus

The advent of immunotherapy for cancer and its acceptance by oncologists has been slow, although momentum is now building as more effective agents are introduced.  Many lessons are being learned and perhaps the most important is that malignant cells are far less autonomous than previously thought. It is now appreciated that the behavior of cancer cells and the prognosis of the underlying disease are critically determined by the tumor microenvironment and, in particular, by elements of the immune system that reflect the ‘immune landscape’ [1]. Indeed, the local inflammatory response has been termed “the other half of the tumor” [2].

Until very recently, emphasis has been placed on primary site TNM staging classification, the microscopic appearance of the tumor (histology and grade), and a few biomarkers, such as ER/PR, HER2, k-RAS, and b-Raf for the establishment of the status and prognosis of a cancer.  Consideration of the reaction of the patient to the cancer has at best been limited to somewhat contradictory comments about lymphocyte infiltration; it has only recently been recognized that the stromal milieu of a tumor is a major component of the host-cancer battle and, accordingly, a marker for prognosis. This concept becomes self-evident if the tumor is seen as an obligate parasite, capable of inducing a chronic inflammatory response and subverting the host's immunity. In the case of colorectal cancer, for example, identification of the subsets of T-cells infiltrating the tumor provide a far better prognostic index than the classical Duke’s staging [3-4].

An indication of the importance of the immune profiles of patients came from two studies based on the use of mRNA profiling to detect altered expression of genes apparently associated with progression and outcome in castration-resistant prostate cancer [5-6]. Although the identified genes in the two studies differed, perhaps due to technical differences, they were, in each case, found (to the authors’ expressed surprise) to be associated with dysregulated immune function rather than with oncogenesis.

## Review

### The tortoise and the hare

Another important recently learned lesson is that immunotherapy works at a different rate to chemotherapy, and the criteria for response need to take this into account. The clinical course of immunotherapy, compared to that of chemotherapy, has been likened to Aesop’s fable of the Tortoise and the Hare [7]. Chemotherapy often induces a rapid reduction in tumor size, followed by re-growth; however, while current immunotherapeutic strategies may lead to a reduction in tumor size, in many cases they do not do so but rather lead to a slowing of progression with ultimately a more favorable course of the disease. Tumor size does not always correlate with survival. Indeed, the tumor may enlarge and new lesions may appear, yet the patient remains relatively well, and the increase in size of the tumor may, at least in part, be the result of infiltration by effective immune cells and the ensuing inflammatory response [8].

Thus, as noted by the Translational Research Working Group of the National Cancer Institute [9], the conventional Response Evaluation Criteria In Solid Tumors (RECIST), version 1.1 (based largely on percentage change in tumor bi-dimensional measurements), is not the best measure of the effects of immune response modifiers; a new methodological framework for the emerging discipline of ‘immuno-oncology’ is required [10-12]. To this end, international collaborative efforts are being made to develop an ‘immunoscore’ to aid the classification and typing of tumors [13].

It is also becoming clear that there are no immunotherapeutic ‘magic bullets’ for cancer and that single pathway assaults are likely to lead to the development of resistance, much as happens with antimicrobial therapy, especially that of tuberculosis [14]. The need for multidrug therapy is not a new concept, as most oncologists will confirm. It is now clear that for maximum efficiency, combinations of immunotherapeutic agents will need to be used with other therapies designed to maximize efficacy. This will include: 1) Surgery to debulk the disease, 2) Physical injury to the tumor in order to release tumor-specific antigens (so-called immunogenic cell death), for example, by hypofractionated radiotherapy, cryotherapy or radiofrequency ablation, 3) Targeting of the subverted inflammatory response induced by the tumor (reversal of the immunosuppressive effects of regulatory T-cells (Tregs) and tumor-associated macrophages, and using immunomodulators and immunoadjuvants to tune and steer an appropriate immune response). 4) Freeing up the host immune response by releasing the ‘brakes’ with the new so-called ‘checkpoint inhibitors’ - anti-PD-1, PD-L1, etc.

It is also becoming clear that each tumor type will need a different therapeutic regimen; it may indeed be that each patient will need further modification based on individual immune function, thus truly achieving personalized therapy.

### Drug repositioning

The purpose of this paper is to highlight what has been termed ‘drug repositioning’, defined as “the utilization of a known compound in a novel indication underscoring a new mode of action that predicts innovative therapeutic options” [15]. The modern oncologist need have no fears that chemotherapy is defunct. It most certainly is not, but it will require adaptation to these new methodologies, and also to take the host's immune status into account.

The era of modern chemotherapy dates its origin to observations on the effects of mustard gas and similar cell poisons. It has become generally accepted that, for maximum efficacy, it is necessary to treat patients to the maximum tolerated dose (MTD) as determined by Phase I/II studies, or the treatment may fail. Thus, the immunosuppressive and lymphoablative properties of chemotherapeutic agents for cancer given at MTD have been accepted as unavoidable if a clinical response is to be achieved, even in extreme cases in which the patient receives a potentially lethal dose of chemotherapy and is then salvaged with a bone marrow or stem cell transplant. Sadly, this approach has met with limited success and is very reminiscent of the old days of treating diabetic ketoacidosis with massive doses of insulin. The outcomes were awful until it was realized that regular infusions of small doses of insulin combined with fluid correction were far more effective [16]. It is time to realize that the MTD concept in cancer chemotherapy may no longer be appropriate in many situations, and that better results may be achieved by using smaller and more regular dosing [17-18]. Better still, this should be combined with other treatment modalities in a rational way in order to maximize the therapeutic effect, as mentioned above.

A principal reason for such drug repositioning is that, in recent years, there has been a paradigm shift in the understanding of the biology of cancer, particularly in the central role of the immune system in eliminating early cancers and allowing those that are not eliminated to exist in a state of equilibrium for varying periods of time [19]. Once the tumors have escaped immune control, dysregulated immune reactivity, manifesting as chronic inflammation [2], aids tumor progression by several mechanisms, including the enhancement of angiogenesis. There is also now strong evidence that regulatory T cells play a key role in tumor-associated local immunosuppression [20]. Most, but not all, Tregs are characterized by the expression of the transcriptional regulator FoxP3, and although CD4^+^, CD8^+^, FoxP3^+^, and FoxP3^-^ Tregs have been described, the one receiving most attention in cancer is the CD4^+^CD25^+^FoxP3^+^ subset which is associated with a poor prognosis in many cancers [21].

A further reason for drug repositioning is that it has been demonstrated that most, if not all, chemotherapeutic agents have beneficial effects on the immune system at low doses [22-23].

### Hormesis

**Figure 1:**
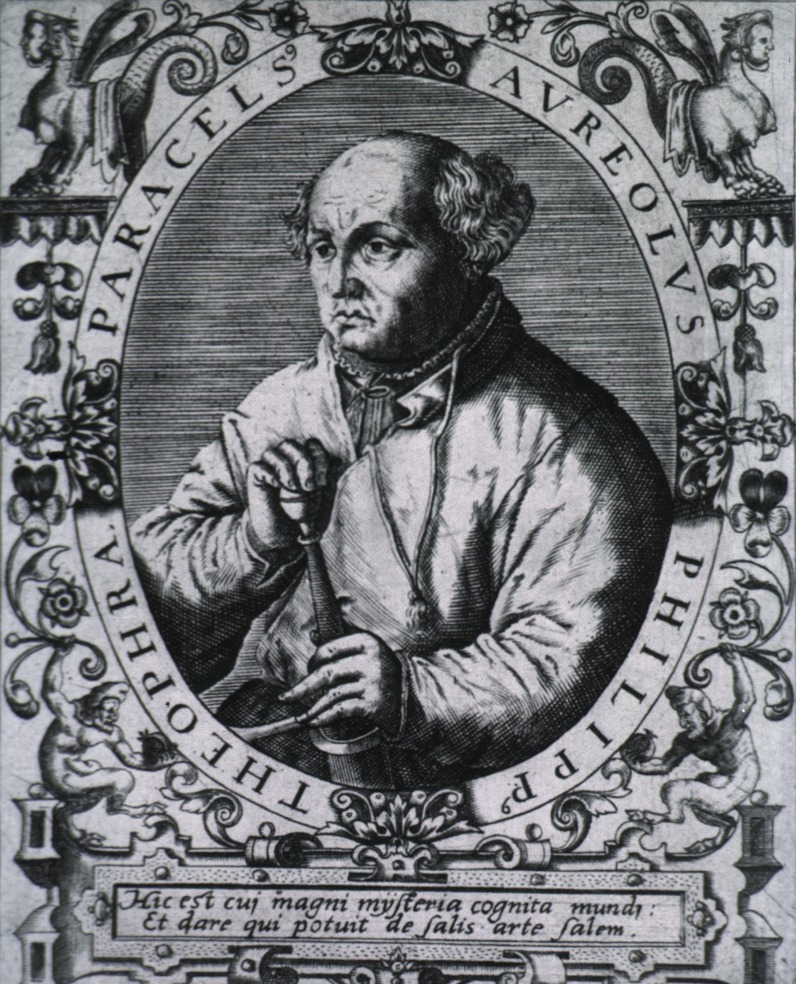
Paracelsus (Theophrastus Bombastus von Hohenheim) 1493-1541 Engraving from life by Augustin Hirschvogel in 1538.

The 16^th ^century physician and philosopher, Theophrastus Bombastus von Hohenheim, known as Paracelsus (Figure 1), stressed the importance of determining the optimal dose of a therapeutic agent as almost all substances are poisonous when administered in sufficiently large doses [24]. The very common phenomenon of a therapeutic agent having a beneficial effect at a low dose and toxic effects at a higher one was subsequently termed hormesis, from the Greek *hormáein* (to set in motion or urge on). The subsequent literature on this phenomenon reveals a lack of clarity in the exact meaning and usage of this expression, not least because the term covers a very wide range of phenomena [25-27].

A special form of hormesis relevant to cancer therapy is that in which agents have radically different effects at high and low doses, but with claims for each having beneficial therapeutic effects. This is in contrast to the usual definition of hormesis in which the higher dose is merely poisonous. In this discussion paper, we consider examples of agents that at high doses have the effects of cytotoxicity and immunosuppression, but at lower doses have alternative beneficial effects operating through the immune system.

### Metronomic chemotherapy

There has been increasing interest in the use of low-dose, well-tolerated, intermittent chemotherapy, termed ‘metronomic’ chemotherapy [28], as an alternative to the conventional high-dose approach.

Cyclophosphamide provides a good example of hormesis and the development of low-dose metronomic chemotherapy (LDMC). At high doses, it exerts its anti-tumor effect solely by cytotoxicity with bone marrow suppression as a potentially fatal adverse effect; at low doses, it contributes to anti-tumor immunity as described below.

At the time of this writing, no Phase III studies of LDMC with cyclophosphamide have been published, but several Phase II studies indicating safety and efficacy have been completed [17, 29]. For example, cyclophosphamide-based LDMC has been evaluated in a randomized Phase II trial involving patients with a range of advanced cancers who had exhausted all effective therapies [30]. Median overall survival in the cyclophosphamide group was 195 days, as compared to 145 in the controls, although there were differences between cancers with much better responses in patients with sarcomas than in those with gastrointestinal cancers. Other workers have reported a similar good response in sarcoma patients [31]. Overall, metronomic cyclophosphamide is well tolerated and provides a period of stable disease in cancer patients with a very poor prognosis and, therefore, warrants further evaluation.

Immune Regulation

Over the last decade, a principal force driving the work on LDMC has been its inhibitory effect on tumor angiogenesis, which is essential for the development of primary and metastatic tumors greater than a few millimeters in size, and is therefore an important target for therapy [32-33]. This anti-angiogenic effect has been demonstrated in animal models and human studies, and the somewhat variable clinical responses to LDMC aimed at suppressing angiogenesis have been reviewed [32].

More recently, however, it has emerged that suppression of angiogenesis is not the only beneficial effect of LDMC – other mechanisms include the restoration of anti-cancer immunity and a return from the state of ‘escape’ to that of ‘equilibrium’ [15, 34]. In this context, LDMC with cyclophosphamide strongly mediates dendritic cell (DC) homeostasis: in murine and *ex vivo* studies, cyclophosphamide and anthracyclines induced a form of apoptosis that released tumor antigens (immunogenic cell death) and strong DC-activating signals [35]. Agents, such as cyclophosphamide and the anthracyclines, with the ability to mediate enhanced immunogenicity form the basis of what has been termed ‘immunogenic chemotherapy’ [36]. Far from causing bone marrow suppression, low dose cyclophosphamide enhances the generation of DC precursors while low dose vinblastine induces their maturation [34].

Other effects of LDMC relevant to beneficial immune responses in cancer include a shift in the cytokine profile from Type 2 to Type 1 [35, 37-38], proliferation and prolonged survival of lymphocytes [39], particularly effector Th1 T cells [15, 35], DC mobilization [40], activation of CD11b myeloid cells, and sensitization of tumor cells to TRAIL-dependent lysis by CD8^+^ cytotoxic T cells [41]. These features all contribute to effective anti-tumor immune response.

By preferentially eliminating Tregs, LDMC alters the Treg/effector T cell balance in favor of anti-tumor effects [42-43]. In a study on patients with therapy-refractory metastatic breast cancer, LDMC with cyclophosphamide selectively eliminated Tregs whilst preserving CD4^+^ and CD8^+^ effector T cells [44]. The decrease in Tregs was only transient but the increase in anti-tumor T cells was stable and sustained, with the number of tumor-reactive T cells (but not that of Tregs) correlating significantly with disease stabilization and overall survival [45]. Other workers have also observed a diminution in Tregs in patients treated with cyclophosphamide-based LDMC and recommend the use of such therapy before commencing a course of immunotherapy [42]. By contrast, it was shown in one study that the reduction in Tregs on commencement of LDMC was not only a short-term event, but was followed by an enhancement of these cells and their immunosuppressive activity [46]. This study [46] emphasized the importance of combining LDMC with immune modulating strategies, which synergize with the chemotherapeutic regimen. Despite this report, metronomic cyclophosphamide was given to patients in a Phase II study of an autologous-pulsed DC vaccine for melanoma. Although it did not indeed lead to a decrease in the number of Tregs, the clinical and immune responses to the antigens used for pulsing the DCs were higher, and the survival longer than in a previous trial without cyclophosphamide [47].

Reduced expression of major histocompatibility complex class I (MHC-I) molecules, resulting in diminished antigen presentation, is one mechanism of tumor escape from immune attack [48]. Both LDMC with several chemotherapeutic agents (including topotecan, etoposide, cisplatin, paclitaxel, and vinblastine) and radiotherapy have been shown to mediate elevated MHC-I expression in cancer cells through induction of IFN-beta [49]. The authors postulate that restoration of MHC-I expression could be an important component of the enhanced cytotoxic T cell activity observed in animals and patients treated with LDMC.

An intriguing example of a combination of induced antigen release from tumors and cyclophosphamide-based LDMC is provided by the administered low-dose cyclophosphamide together with intratumoral injection of an oncolytic adenovirus to patients with advanced and progressive cancer unresponsive to conventional therapy. This therapy led to an increase in numbers of cytotoxic T cells, a systemic Th1 pattern of immune reactivity, a decrease in Tregs, a significantly improved control of disease in comparison to virus alone (p<0.0001), and unusually high progression-free survival and overall survival for patients refractory to standard chemotherapy. It is probable that the oncolytic virus liberated tumor antigens to ‘feed’ the immune system, or perhaps provided danger signals so TAA's released by chemotherapy could be effectively dealt with.

### The microbiome

Another emerging factor requiring consideration is the growing evidence that the micro-organisms on and in the human body – the microbiome – especially the intestinal component, has profound regulatory effects on the immune system [51], and also on the qualitative nature of immune responses against cancer [52]. The intestinal microbiome affects the anti-cancer immunological effects of cyclophosphamide, including the induction of immunogenic cell death, subverts Tregs, and promotes populations of Th1 and a specific “pathogenic” subset of Th17 cells (pTh17) that inhibit cancer growth. In a mouse model, cyclophosphamide also caused inflammation and increased permeability of the intestinal mucosa, allowing commensal bacteria, especially Gram-positive enterococci and lactobacilli, to cross the mucosal barrier and to access the mesenteric lymph nodes and spleen, with a corresponding reduction in the numbers of these bacteria in the gut lumen [53-54].

The role of Gram-positive bacteria in the promotion of Th1 and pTh17 cells was confirmed by treatment of the mice with vancomycin, an antibiotic specific for Gram-positive bacteria [53]. Treatment with vancomycin and, to a lesser extent, other antibiotics including colistin (specific for Gram-negative bacteria) interfered with the ability of cyclophosphamide to inhibit the growth of various tumors, partly by allowing the outgrowth of certain commensals that compromised the anti-cancer effects. This key study provides additional reasons for using cyclophosphamide in low doses, thereby avoiding neutropenia and severe mucositis – two conditions requiring the use of antibiotic therapy. It also emphasizes the importance of avoiding the administration of antibiotics, if possible, during cancer chemotherapy and the need for more research on procedures for beneficially modifying the gut microbiome.

The general conclusion of these studies is that LDMC is a quantum leap forward, but they also implicitly or explicitly emphasize the need for its use together with an immune modulating agent with effects on the immune system that could synergize with those of LDMC.

### Hormesis and the mammalian target of rapamycin

Another class of agents with radically different and opposing effects when used in continuous high doses, and in low and/or intermittent doses, includes rapamycin (sirolimus) and related compounds used for the therapeutic induction of immunosuppression in post-transplant patients. At first view, it would appear a rational assumption that an agent preventing the rejection of a ‘foreign’ transplanted organ would likewise prevent the immune rejection of a tumor but, paradoxically, recent studies have demonstrated immune enhancements and anti-tumor effects at lower doses of rapamycin [55].

The mammalian target of rapamycin (mTOR) is a serine/threonine kinase that has been defined as a “major intersection that connects signals from the extracellular milieu to corresponding changes in intracellular processes” [56]. As such, it integrates a wide range of growth factors and hormonal signals and is a key regulator of cell growth and proliferation [57-58].  Inappropriate activation of mTOR is a prime factor in oncogenesis and angiogenesis [59], as well as the resistance of tumors to chemotherapeutic agents. In addition to cancer, there is interest in the pathogenic role of mTOR in obesity, diabetes, and autism [56]. In this context, it is also noteworthy that inhibition of mTOR by rapamycin reverses impaired social interaction in mice with a tuberous sclerosis-like condition [60] and abolished cognitive deficits and reduced amyloid-beta levels in a mouse model of Alzheimer's disease [61]. Indeed, mTOR appears to be involved in many conditions characterized by chronic inflammation (the manifestation of dysregulated immune reactivity) and is, for example, elevated in human inflammatory bowel disease (IBD). Mouse models of IBD have therefore been used to study the role of dysregulated mucosal immunity in tumorigenesis [62]. Accordingly, there is considerable interest in targeting mTOR, and several agents have been evaluated including rapamycin itself and related compounds, such as everolimus, ridaforolimus, and temsirolimus.

Furthermore, mTOR plays a key role in regulating high energy-requiring T cell differentiation and functional activity [63-64]. In particular, it has a role in the differentiation of CD4^+^ T cells into inflammatory and regulatory subsets, in the induction of anergy, in the development of CD8^+^ memory T cells, and the regulation of T cell trafficking [63]. mTOR also regulates many aspects of the innate immune system [65]. Thus, for example, inhibition of mTOR by rapamycin promoted pro-inflammatory cytokines but blocked the release of IL-10 via the transcription factor STAT3, and rapamycin-treated monocytes displayed a strong Th1 and Th17 cell-polarizing potency. In this context, mTOR mediates the reprogramming of Tregs (a far more plastic population of cells than generally recognised) into functional Th1 and Th17 cells which, depending on context, may be beneficial or harmful with this effect being blocked by rapamycin [66].

From the standpoint of cancer immunotherapy, the effect of rapamycin on DCs is of particular interest. It was shown that a brief exposure of DCs to rapamycin at the time that they are responding to TLR agonists resulted in an extended life span of the DCs and prolonged and increased expression of costimulatory molecules. In turn, this resulted in a particularly potent activation of naïve CD8^+^ T cells and, in vivo, to enhanced control of B16 melanoma in an autologous therapeutic vaccination model in the mouse [67].

Furthermore, from the same standpoint, tumor-associated macrophages play a key role in cancer biology with those associated with Type 2 immunity (M2 macrophages) being associated with angiogenesis and other factors favoring tumor progression. mTOR activation mediates a differentiation into M2 macrophages while treatment with rapamycin induces differentiation into M1 macrophages associated with anti-tumor responses [68].

Rapamycin and several other mTOR inhibitors have been approved for use in therapeutic trials. Several have been conducted, notably in breast cancer, together with various chemotherapeutic regimens or monoclonal antibody-based immunotherapy with encouraging results, although, as stated in a recent comprehensive review [58], more work is required to define their role in therapy. Mouse models for the study of the effect of rapamycin on breast cancer [69] and melanoma [70] have been described and have proved useful for determining biomarkers. Establishing the optimal dose of rapamycin will be critical to its efficacy as its beneficial effects must be balanced against its immunosuppressive properties, and achieving the optimal dose has been likened to adjusting a rheostat [71].

### The ‘repositioning’ of radiotherapy

Radiotherapy treatment may also soon require ‘repositioning’ as concepts of its mode of action are changing from that of a local treatment with the sole intention of causing cell death by damaging DNA through oxidative effects and DNA strand breaks, to alternative modes of action, including effects on the tumor microvasculature and potentiating of anti-cancer immune response. Therefore, there may be a role for ‘radioimmunotherapy’ based on a combination of radiation and immune modulators [72-75]. Clinical trials are in progress. Thus, radiation may have important systemic effects in addition to its local actions.

This change of concept introduces a further aspect of hormesis, one in which a higher dose rather than the lower may have the desired therapeutic effect, but with protection of normal cell tissues by a precise directed limitation of the radiation to the tumor.

Ionizing radiation has the ability to convert the irradiated tumor into an ‘immunogenic hub’ – acting in effect like an autologous tumor ‘vaccine’ [72]. In contrast to conventional (1.8 – 3 Gy) fractionation, high-dose radiotherapy (>8 – 10 Gy per fraction) leads to endothelial cell apoptosis and consequential microvascular dysfunction, which in turn leads to increased cell death. Hypoxia resulting from standard fractionated radiotherapy results in a burst of pro-angiogenic activity in the tumor microenvironment, generating HIF-1α, VEGF, and other vasculogenic factors that then can attenuate radiation-induced apoptosis in endothelial cells. Radiation exposure can provide a source of antigen that is well suited for cross-presentation to the host DCs, which then can induce an antigen-specific immune response [75].

Most irradiated cells survive, at least for a limited time, during which time they undergo a stress response, transmitted through multiple signal transduction pathways to the surrounding tissue. This process is associated with changes in the expression of certain genes, depending on the tissue of origin, the genetic background of the host, the p53 status of the tumor, and the type and regimen of radiation used. Among genes that are up-regulated post-radiation are those controlling expression of growth factors, cytokines, chemokines, and cell surface receptors that modulate the interaction of the tumor with the immune system [76-77].

In addition to excellent local control of disease, high-dose per fraction radiotherapy - stereotactic body radiotherapy (SBRT) - also appears to impact disease outside the irradiated volume. This is best exemplified by the retrospective series from William Beaumont Hospital in which a comparison was made between patients who were treated with either a lobar resection or SBRT during the same time period [78]. SBRT not only resulted in a drastically lower local failure rate (5% versus 24%, p=0.05), but also had a lower regional lymph node failure rate (5% versus 29%, p<0.05). As the patients treated by SBRT had only a very small volume of tissue irradiated (tumor, plus a small margin) and few, if any, lymph nodes were included in the treatment field, this was a surprising finding.

This is likely to be an example of the abscopal effect, from the Greek *ab skopos* – away from the target – resulting from the stimulation of T-cell immunity by tumor antigens released by SBRT, leading to the eradication of occult regional micrometastases. In contrast to SBRT, minimally invasive surgery and open thoracotomy are associated with transient postoperative decreases in circulating CD8^+^ T-cells [79], which may contribute to the increased incidence of regional failure observed with wedge resection compared with SBRT. The lower rate of regional nodal failures after SBRT may be due to increased CD4^+^ and CD8^+^ T-cell immunity.

After radiation exposure, the type of death among the cells programmed to die is highly variable, spanning from apoptosis and necrosis to autophagy and mitotic catastrophe. Importantly, radiation has been shown to induce an immunogenic cell death (ICD), characterized by three molecular signals that promote uptake of dying cells by DCs, cross-presentation of the tumor-derived antigens to T cells and activation of anti-tumor T cells, exposure of calreticulin on the tumor cell surface, release of high-mobility group protein B1 (HMGB1), and release of ATP [80].

A comparison of three SBRT radiation regimens, 20 Gy × 1, 8 Gy × 3, and 6 Gy × 5, demonstrated marked differences between the single dose and the fractionated regimens in the ability to synergize with anti-CTLA-4 antibody treatment and induce an anti-tumor immune response [81]. All three regimens were similar in their ability to cause delayed growth of the irradiated tumor without affecting the growth of a tumor outside the irradiated field. Anti-CTLA-4 by itself or in combination with a single 20 Gy dose was ineffective, but when combined with the two fractionated regimens, it significantly improved inhibition of both the irradiated area and tumors outside the irradiated field. The effectiveness of the generated anti-tumor response was highest with 8 Gy × 3, with 80% of the irradiated tumors and 40% of the tumors outside the field regressing completely. Since anti-CTLA-4 antibody is known to be ineffective against poorly immunogenic tumors but to synergize with vaccination in inducing anti-tumor immunity, these data imply that radiation used as single dose of 20 Gy failed to convert the tumor into an *in situ* vaccine. These results suggest that, for the combination with anti-CTLA-4, there may be an optimal window for the pro-immunogenic effects of radiation, with a hypofractionated regimen providing the best results. Specifically, significant induction of low-density lipoprotein (LDL)-enriched ceramide, secretory sphingomyelinase (S-SMase), tumor necrosis factor-related apoptosis-inducing ligand (TRAIL), and TNF-α in serum from patients treated with SFGRT suggests these bystander effects may have a role in overall tumor response. In view of these encouraging results, the combination of SBRT and immunotherapy in humans is currently being investigated in several studies. 

## Conclusions

The claim that “The human body has no cancer-fighting capabilities”, voiced at the foundation of the German Cancer Research Institute in Heidelberg in 1965 [82], is certainly no longer tenable. The major shift in emphasis is now towards one of seeing cancer as a systemic disease requiring treatment of the host as well as the cancer. This may also involve a repositioning of surgery, radiotherapy, and chemotherapy.

In light of the specific forms of hormesis discussed in this paper, a repositioning of anti-cancer agents, radiation therapy, and the use of combinations of carefully determined metronomic low doses of chemotherapeutic drugs, focused radiation therapy, mTOR inhibitors, and immunotherapeutic agents that modulate the immune system to achieve optimum anti-tumor activity may well prove to be the way forward in the successful therapy of a wide range of cancers. 
